# Rare diseases: ethical challenges in the era of digital health

**DOI:** 10.3389/fdgth.2025.1539841

**Published:** 2025-07-09

**Authors:** Liesbeth Siderius, Sahan Damsiri Perera, Lina Jankauskaite, Anjan Bhattacharya, Paulo Gonçalves

**Affiliations:** ^1^ Youth Health Care, Almere, Netherlands; ^2^ Rare Care World Foundation, Loosdrecht, Netherlands; ^3^ Rosemary Bryant AO Research Centre, University of South Australia, Adelaide, SA, Australia; ^4^ Department of Paediatrics, Medical Academy, Lithuanian University of Health Sciences, Kaunas, Lithuania; ^5^ Coordinating Centre for Rare and Undiagnosed Diseases, Lithuanian University of Health Sciences Hospital Kauno Klinikos, Kaunas, Lithuania; ^6^ Child Development Centre, Apollo Multispeciality Hospital, Kolkata, India; ^7^ West Bengal University of Technology, Kolkata, India; ^8^ RD-Portugal, Lisboa, Portugal

**Keywords:** rare diseases, public health, person-centred care, digital health, data interoperability, philosophy, artificial intelligence in medicine, Shwachman-Diamond syndrome

## Abstract

To improve the health and wellbeing outcomes of people with rare conditions, it is necessary to integrate all aspects of health and wellbeing. Digital health technologies can appropriately capture and share harmonised data between care providers and the individuals concerned. The quality of digital health is dependent on defined data points reflecting the actual medical and societal situation and register changes when new diagnostics or therapies become available. The life experiences of individuals living with a condition, individually or as a group, are underrepresented in the digitalising world. This narrative review addresses rare conditions as an entity, public health strategies, digital health opportunities, and ethical considerations. The challenge is illustrated by comparing data gathered by manually selected data points with advanced artificial intelligence systems. In this new digital era, we consider the philosopher Kant's notion of noumena: “Only individuals with rare disabling conditions can genuinely convey the reality of living with those conditions”. In conclusion, there is a pressing demand to embed the needs and experiences of people in all new technologies.

## 1 Introduction

The general public, including decision-makers, is usually not aware of how it is to live with a chronic, disabling and rare condition unless by personal experiences. This general lack of awareness leads to health and social care system deficiencies. Consequently, these gaps result in deficiencies in health and social, integrated, holistic, real-life digital data. Rare conditions are defined as affecting fewer than 1 in 2,000 people. More than 6,000 such diseases have been identified. Many of the rare conditions are genetic in origin and manifest in childhood (1). The exploding knowledge of human genetics over the last 70 years in the upcoming digital era is viewed in light of human rights and person-centred care. The primary aim of this narrative review is to emphasise the importance of integrating personal experiences in digital health frameworks, focusing on real-life data for rare, chronic, and disabling conditions. The case of Shwachman Diamond Syndrome (SDS) illustrates obstacles to be aware of before jumping to artificial intelligence without properly defining quality digital data. Recommendations are provided on how the voices of an individual can be integrated into digital communications.

## 2 Methodology

Because of the conceptual complexity of the research topic, a narrative review was performed to provide an overview of the evidence available. As science is partial, we navigate through sub-topics representing different aspects composing the addressed aim, illustrated with tartgetted literature and policy analysis. There may be an inordinate weight on some sub-topics identified for readers already familiar with the issue, where others are not aware of:

- The introduction of rare diseases as an identity (1).
- Public Policies: A selection of relevant public health strategies addressing rare diseases are reviewed (2).
- Technical considerations: A literature survey on digital health opportunities in relation to interoperable medical data (3).
- A case study on data points: integrating health and social data points on an international guideline Shwachman Diamond Syndrome was abstracted (4).
- AI data comparison: Manually selected data points for digital exchange were compared with data collections by ChatGPT (5).
- Ethical challenge: European law brought concerning Kant's notion of noumena (6).

### 2.1 Rare diseases as a new identity

Over the past decades, the field of medicine has experienced revolutionary advancements, beginning with the discovery of deoxyribonucleic acid (DNA)'s double-helix structure in 1952 by James Watson and Francis Crick, together with noteworthy contributions from chemists Rosalind Franklin and Maurice Wilkins, who demonstrated how the structure of DNA encodes proteins (2). In 1959, researchers established a link between clinically defined conditions, such as Down syndrome, and numerical chromosome abnormalities (3). This achievement was succeeded by the molecular mapping of Huntington's disease, an autosomal dominant disorder, to a specific human chromosome, marking another significant breakthrough in medical genetics (4). To connect these advancements to the current digital landscape, it is crucial to explore how these discoveries have influenced data structures and interoperability in healthcare.

Currently, we have identified thousands of different chronic conditions that arise from changes in our genetic material. Individuals living with these rare conditions face common diagnostic challenges and unmet medical and social needs. They often encounter diagnostic failures, uncertainty about treatment options, and various disabilities that affect their health, psychosocial wellbeing, and economic circumstances. Rare Diseases Europe recently reported the impact of living with a rare disease (5). 10,478 people living with a rare disease and family members worldwide, with more than 1,600 distinct rare, participated in the survey. While 8 out of 10 people with rare diseases live with disabilities, their disabilities were not sufficiently recognised. Unacceptable is that 58% experienced discrimination related to the rare disease or disability in healthcare, housing, employment and education.

A robust digital framework incorporating genetic, clinical, and social data will enhance early interventions, integrate health management and optimise full societal integration.

For instance, integrating kidney monitoring protocols for tuberous sclerosis-associated renal angiomyolipoma (6) and routine bone marrow surveillance for fatal bone marrow failure in the case of Shwachman Diamond syndrome (7) into digital health systems can provide early warnings for potential complications. Although these examples differ, both represent the commonalities of rare diseases: multiple clinical diseases caused by one genetic diagnosis. This approach exemplifies how a single clinical intervention can prevent severe health outcomes, yet it primarily addresses clinical endpoints without capturing person-reported outcomes that could inform digital frameworks. A historical perspective further illustrates this disconnect: in the 1960s, the success of dietary interventions in phenylketonuria infants sparked significant public interest, leading parents of children with intellectual disability to advocate for routine newborn screening through a simple blood test. This advocacy laid the groundwork for the widespread implementation of newborn screening programs using dried blood spots, underscoring how clinical data collection can evolve into broader public health frameworks^.^

A logical approach to digitising healthcare is to organise individual health and social data based on inherited DNA variations. Genome-wide association studies have provided significant insights into the pathobiology of many diseases; however, these insights have not yet translated into substantial improvements in healthcare. Researchers tested a dataset of primary participants of European ancestry for five diseases. Diagnosis of prevalent disease was based on a composite of data from self-reports in interviews with a trained nurse and electronic health record (EHR) information, including the inpatient International Classification of Diseases (ICD-10) diagnosis code. A familial hypercholesterolemia mutation in 0.4% of the population gives an up to 3-fold increased risk for coronary artery disease. For various common diseases, genes have been identified in which rare mutations confer several-fold increased risk in heterozygous carriers. The authors suggest we may soon reach a point where individual whole genome variant profiling can effectively assess disease risks as part of routine healthcare (8). Initiatives are underway to perform newborn genome sequencing to develop a comprehensive framework for future healthcare integration (9).

Identifying persons with rare diseases with new techniques available opens the way to providing adequate support for full participation in daily life.

### 2.2 Assessment of public policy

Public health focuses on preventing illnesses, injuries, or deaths, protecting vulnerable populations, and promoting policies and actions that enhance health and safety. For example, physically screening newborns within their first weeks using the red light reflex method can help identify individuals at high risk for conditions like congenital cataracts and prevent lifelong visual impairment (10). Scientists, politicians, and the general public were excited by the outcomes of diet-treated phenylketonuria infants in the 1960s. Parents of children with intellectual disability began to advocate to test all newborns by a simple blood test. The gateway to the worldwide newborn screening on dried blood spots (11).

The United Nations has adopted a resolution to promote the integration of rare diseases into its agenda, reinforcing its commitment to achieving the 2030 Sustainable Development Goals of “leaving no one behind” (12, 13). The United Nations (UN) General Assembly has emphasised the key role of universal health coverage (UHC) in achieving health for all. UHC is, therefore, a centrepiece of the health-related Sustainable Development Goals (SDGs) (14, 15).

On 24 May 2025, Member States of the 78th World Health Assembly adopted the Resolution on Rare Diseases: a remarkable milestone in the global effort to improve the lives of the over 300 million people living with a rare or undiagnosed disease (16). As part of the resolution the WHO urges members states to commit “to promoting the involvement of patient organizations, peer support groups, organizations of persons with disabilities, including groups led by persons living with a rare disease, in policy development to ensure that the voices of those affected by rare diseases are heard and incorporated into decision-making processes”.

The 78th World Health Assembly also approved the extension and renewal of the Global Strategy on Digital Health to improve health care systems with greater attention to individual needs and leave no one behind.

Major steps in digital health have been taken. In January 2025, the Indian Ministry of Health and Family Affairs of India posted: From Data to Diagnosis Transforming Healthcare through Digitalisation. Indicating India's Ayushman Bharat Digital Mission and the Digital Health Incentive Scheme can set a global benchmark for digital healthcare transformation (17). On Mar 26, 2025, the European Health Data Space entered force. “It enhances individuals” access to and control over their electronic health data while enabling certain data to be reused for public interest, policy support, and scientific research purposes’ (18).

However, many existing public health policies do not adequately account for the unique needs of rare disease populations, particularly in integrating patient-reported outcomes into digital frameworks.

They are depending on experiences from people living with the disease. So what do people with rare and disabling conditions need to know about digital transformation?

### 2.3 The upcoming era of digital health

Digital technologies have great potential to strengthen health systems and improve access to healthcare, particularly in rural and remote communities (19). Digitalisation in healthcare can accelerate early diagnosis, put the person at the centre of disease management, and provide access to specialised social care for patients and their families. The success of digital health initiatives largely depends on the ability to capture stratified data that can be analysed to enhance decision-making. However, the effectiveness of digital health initiatives is contingent upon integrating high-quality, interoperable data, which is often lacking in the context of rare diseases (20).

Moreover, while data from routine care can potentially reduce unavailable data often lacking in randomised clinical trials and observational cohort studies, these data must be harmonised to ensure accuracy and reliability (21). Integrating person-reported outcomes is crucial in this context, as it provides insights that extend beyond clinical endpoints, encompassing quality of life, social participation, and daily functioning, especially in rare disease populations.

Disease phenotypes and management guidelines are documented in various online peer-reviewed published resources. Extracting interoperable data points from these texts requires manual effort, which can be labour-intensive and biased based on the clinician's background and training. This underscores the importance of structured data frameworks that can effectively capture diverse data points while maintaining semantic consistency. Collaboration among multiple disciplines is necessary to address the diversity, complexity, and specificity of data sequences of rare diseases.

Standards-based, open-source clinical decision support systems (CDS) are designed to represent knowledge and facilitate validation. The earliest standard in this field, the Arden Syntax for Medical Logic Systems, has since become a Health Level Seven International (HL7) standard (22). HL7 promotes data interoperability between EHRs. Stakeholders can create Fast Healthcare Interoperable Resources (FHIR) profiles by utilising HL7 resources. These profiles assign semantic values from standardised vocabularies to the characteristics of various resources, enabling seamless bidirectional communication between EHRs. An example of a standard vocabulary important for people with inherited neutropenia is the laboratory test Neutrophil Count converted to the interoperable standard Logical Observation Identifiers Names and Codes (LOINC): LOINC - 26499-4- (Table 1).

**Table 1:** Manual/chat.

Chat GTP	Comment
Bone Marrow Biopsy - LOINC: 721-1 (Bone marrow biopsy interpretation)	Wrong Code: 721-1 Free Hemoglobin [Mass/volume] in Plasma. Correct code could be LOINC - 33721-2, that is Bone marrow Pathology biopsy report
Complete Blood Count (CBC) - LOINC: 6690-2 (WBC, Platelets, Hemoglobin, Hematocrit, etc.)	Wrong Code: 6690-2 Leukocytes [#/volume] in Blood by Automated count. Correct code could be 58410-2, that is CBC panel - Blood by Automated count which is the traditional heamogram, platelet count and leucocyte count Alternatively, LOINC - 26499-4- Neutrophils [#/volume] in Blood [value]10 × 3/ul, LOINC - 718-7 Hemoglobin [Mass/volume] in Blood [value]g/dl, LOINC - 777-3 Platelets [#/volume] in Blood by Automated count [value] × 1,000/ul Could be used to monitor the three lineages of the blood
Neutrophil Count - LOINC: 26450-5 [Neutrophils (#/volume) in Blood by Automated count]	No Such Code. The correct code is LOINC - 26499-4- Neutrophils [#/volume] in Blood [value]10 × 3/ul as mentioned above
Reticulocyte Count - LOINC: 30385-9 [Reticulocytes (#/volume) in Blood by Automated count]	Wrong Code: 30385-9 Erythrocyte distribution width [Ratio] Correct code could be 14196-0 Reticulocytes [#/volume] in Blood. This should be interpreted with the heamoglobin level as in SDS anaemia with low reticulocytes occurs
Fecal Elastase - LOINC: 2724-8 [Elastase (Enzymatic activity/volume) in Stool]	No Such Code. The correct code could be LOINC - 25907-7 Elastase.pancreatic [Mass/mass] in Stool ug/g
Fecal Fat Test - LOINC: 2965-2 [Fat (Mass/volume) in Stool]	Wrong Code: 2965-2 Specific gravity of Urine The correct code could be 32545-6 Fat [Moles/time] in 72 h Stool. As the requirement is to know the fat balance Fat intake (32544-9 Fat intake 72 h) is also needed to be measured.
LOINC: 82122-9 (SBDS gene mutation analysis in Blood or Tissue by Molecular genetics method)	No Such Code. Correct code could be LOINC - 41764-2 - SBDS gene-targeted mutation analysis in Blood or Tissue by Molecular genetics method
LOINC: 1920-8 [Aspartate aminotransferase (Enzymatic activity/volume) in Serum or Plasma]	Correct Code
LOINC: 1742-6 [Alanine aminotransferase (Enzymatic activity/volume) in Serum or Plasma]	Correct Code
LOINC: 14629-0 [Vitamin D (Mass/volume) in Serum or Plasma]	Wrong Code: 14629-0 Bilirubin.direct [Moles/volume] in Serum or Plasma. The correct code could be 14635-7 25-hydroxyvitamin D3 [Moles/volume] in Serum or Plasma (Calcidiol) which is the Vit D derived from animal sources
LOINC: 14647-2 [Vitamin A (Mass/volume) in Serum or Plasma]	Wrong Code: 14647-2 Cholesterol [Moles/volume] in Serum or Plasma. Correct code could be 14905-4 Retinol [Moles/volume] in Serum or Plasma

HL7 FHIR inherently supports RESTful application programming interfaces, which follow the design principles of the representational state transfer (REST) architectural style. Therefore, FHIR defines a healthcare data model as a collection of RESTful services for exchanging healthcare data between applications. This approach provides opportunities for broader dissemination and sharing of CDS tools, including a resource category for mapping outputs in different natural languages.

The HL7 FHIR healthcare data model is not limited to data describing individual patients. Instead, FHIR interoperability solutions are composed of modules called “resources”, which are small, reusable components that define a set of properties to capture the structure of domain data for specific uses. This modular approach is particularly beneficial for rare disease populations, as it creates specialised data profiles that can capture disease-specific nuances without sacrificing interoperability.

Amar et al. conducted a systematic mapping review using ten electronic databases to analyse research published between 2012 and 2022 on semantic interoperability using the FHIR standard (23). Terminologies were often mapped to Systematised Nomenclature of Medicine Clinical Terms (SNOMED CT) and LOINC, which appeared in more than half of the selected studies. SNOMED CT was primarily used for medication, procedures, and conditions (problems) and diseases and diagnoses International Classification of Diseases (ICD)-10 and SNOMED CT. LOINC was used for observations and diagnostic reports. Examples for practical use: SCTID: 271700006 Chorea (disorder) and for more specifically Huntington's chorea (disorder) SCTID: 58756001 and LOINC 46746-4 for phenylketonuria and variants/Biopterin defects newborn screen interpretation.

However, these terminologies primarily focus on clinical endpoints, often neglecting person-reported data such as the International Classification of Functioning, Disability and Health (ICF) and LOINC's uniform representation of Social Determinants of Health data elements. Both are crucial for a holistic understanding of rare disease outcomes.

The ICF in EHR's Health was systematically reviewed (24). Maritz et al. concluded that the ICF is not structured as a formal terminology. A reduced number of ICF codes for more feasible and practical use is recommended, such for example, on housing (ICF-d610 acquiring a place to live), on employment (ICF-d850 remunerative employment) and on education (ICF-d820 school education) (5). Integrating ICF codes within FHIR resources could enhance data frameworks by providing a structured representation of functional status and social participation, bridging the gap between clinical data and real-world patient experiences.

The World Health Organization developed the Digital Adaptation Kits within the SMART (Standards-based, Machine-readable, Adaptive, Requirements-based, and Testable) guidelines to evolve guidelines-based data dictionaries by mapping to HL7/FHIR and semantic terminology (25, 26). These implementation guides display data dictionary content as FHIR resources and semantic terminology codes. Harmonising data collections generated from evidence-based medical guidelines provides a platform for monitoring the complex dynamics of healthcare using Real World Data, from diagnosis to treatment and participation in society, creating a more comprehensive and person-centred data ecosystem (27).

Researchers have questioned whether the clinical content of a dataset specifically developed for implementation in FHIR can also be represented using openEHR archetypes during the COVID-19 pandemic (28). OpenEHR is a public library of standards-based, vendor-neutral clinical information models that can be reused across various health datasets. The findings support the openEHR hypothesis that creating a shared, public library of standards-based, vendor-neutral clinical information models can be reused across a diverse range of health data sets is possible. Even though the focus and level of detail for each template varied, the shared data models illustrated each template and ensured data consistency across all of them. This flexibility is particularly relevant for rare diseases, as it enables the development of customised templates that can capture disease-specific data points while ensuring compatibility with broader FHIR frameworks.

Natural language processing can help automate data extraction. However, developing a digitally interpretable linguistic framework from written text requires human involvement. Large language models (LLMs), such as OpenAI's Chat Generative Pre-Trained Transformer (ChatGPT-4), are advanced AI systems trained on extensive amounts of existing text. Nevertheless, the sources of this information remain unclear. These models can automatically identify disease-related concepts, but the challenges in understanding contextual meaning may limit their effectiveness. Therefore, clinicians must critically evaluate the outputs of these models, especially when defining essential data points. Furthermore, all stakeholders need to be aware of the limitations of LLMs when considering them as tools to support the diagnosis and treatment of rare diseases (29).

### 2.4 Case study integrating medical and social data points

Although there are over 6,000 rare conditions, people with rare conditions experience common diagnostic challenges and unmet medical and social needs. The complexity and variability of rare diseases present significant obstacles to data standardisation, which is essential for digital health integration. Each rare condition can be transferred into FHIR profiles with properly assigned semantic values from standardised vocabularies, enabling seamless bidirectional communication between EMRs. However, the absence of universally accepted data frameworks for rare diseases complicates data integration, making it difficult to ensure consistency and interoperability across diverse health systems.

Interoperable real-life data enhances communications among academic specialists, general health providers, social services, and the persons concerned. By structuring data in standardised formats such as HL7 FHIR, health systems can enable effective data exchange that mitigates diagnostic delays and reduces the risk of fatal complications.

The case study on Shwachman Diamond Syndrome is an example of how -in general- a guideline can be transformed to be adapted for Digital Health Integration using standardised international codes and classifications (27). SDS is a rare autosomal recessive disorder characterised by exocrine pancreatic insufficiency, bone marrow dysfunction, skeletal abnormalities, and autistic-like behaviour. SDS ranks as the second most common cause of inherited pancreatic insufficiency after cystic fibrosis, as well as the third most common inherited bone marrow failure syndrome, with an elevated risk of mortality. Approximately 90% of SDS cases are linked to mutations in the SBDS (Schwachman-Bodian-Diamond syndrome) gene on chromosome 7, which was discovered in 2003. Clinicians have widely utilised the Draft Consensus Guideline for diagnosing and treating Shwachman-Diamond syndrome (30) to manage SDS patients. As an example of a complex rare disease, we systematically transformed the SDS clinical guideline into software-neutral specifications, terminologies, and interoperability standards. This process involved several key steps.

First, we identified and delineated pivotal data points within the guidelines to ensure a comprehensive understanding of the information to be encoded. Next, we searched for suitable terminologies and adapted them to accurately represent and categorise these data points, using SNOMED CT, LOINC, ICD, and ICF to lay the foundation for effective communication within digital health systems. Finally, to promote widespread implementation and collaboration, the profile is accessible through https://www.simplifier.net, a dedicated platform designed to facilitate the sharing and dissemination healthcare standards and resources. This structured approach facilitates clinical data integration and creates pathways for integrating social and personal-reported data. In the case of SDS, digital frameworks can be expanded to include social determinants of health, functional status assessments, and quality-of-life indicators using the ICF model. This inclusion is particularly relevant given the diverse impacts of SDS, ranging from pancreatic insufficiency to developmental delays and social participation limitations.

Depending on various personal factors and environmental conditions, SDS significantly impacts an individual's ability to participate in society. To conduct a thorough assessment of disabilities while managing the disease, various social aspects can be evaluated. The ICF is the WHO framework for measuring health and disability at both individual and population levels. The ICF details an individual's functioning from a bio-psycho-social perspective (31, 32). It also appears in the HL7 list of terminologies. Consequently, a person's strengths and weaknesses—providing a basis for individualised recommendations for social intervention—can be included in EMRs and personal health records for sharing with others. Developing condition-specific core sets within the ICF framework helps translate social and functional data into standardised formats suitable for inclusion in EMRs, personal health records, and interoperable FHIR resources (24).

### 2.5 FHIR profile manually vs. ChatGPT's

Regarding the issue of the interoperability of data sets and the use of artificial intelligence (AI), we designed one use case, Shwachman Diamond Syndrome. This approach aimed to assess the reliability of AI-generated outputs compared to established clinical guidelines. When prompted with the question, “What are the features of Shwachman-Diamond Syndrome?” ChatGPT-4 provided a general overview. It defined SDS as “a rare inherited disorder that affects multiple systems in the body, particularly the bone marrow, pancreas, and skeletal system”. This demonstrates AI's capability to summarise established medical knowledge. However, limitations became evident in areas requiring nuanced, condition-specific details.

Regarding diagnostic methodology, ChatGPT-4 described a combination of clinical evaluation, laboratory tests (including blood counts and pancreatic enzyme levels), genetic testing, and bone marrow biopsy. While this information was largely consistent with the SDS guidelines, the AI omitted key diagnostic elements, such as radiological findings, which are integral to the comprehensive diagnostic workup for SDS. This underscores a critical gap in AI-generated content's inability to consistently incorporate detailed, condition-specific diagnostic criteria beyond standard clinical descriptors. However, the SDS guideline also includes radiological findings in their diagnostic workup, emphasising that genetic testing is confirmatory. The ChatGPT states that the management of SDS often involves a multidisciplinary approach, including haematology, gastroenterology, endocrinology, and other specialities, to address the various complications associated with the syndrome, consistent with the management described in the SDS guideline. However, ChatGPT failed to provide information on perceived social participation and functional assessments, which are crucial for holistic care in rare disease populations and align with the ICF model.

When ChatGPT-4 was questioned about the LOINC codes necessary for capturing patient data related to SDS (Table 1), the AI provided 11 codes, but only two were correct, demonstrating a phenomenon known as AI hallucination. AI hallucination or misinformation occurs when a LLM, such as ChatGPT, Google Bard, or Microsoft AI Sydney, generates patterns or outputs that are non-existent or imperceptible to human observers, resulting in nonsensical or inaccurate information (33).

Utilising these “hallucinated” codes generated by language models poses critical risks to data interoperability, as the incorrect codes would be unrecognisable to recipients. This misalignment could cause substantial problems in clinical data exchange and integration, undermining the reliability and effectiveness of EMRs and other health information systems. Hence, while LLMs like ChatGPT offer valuable insights, their outputs must be rigorously validated to prevent potential disruptions in healthcare interoperability.

LLMs' limitations also extend to obtaining informed consent, a crucial component for patient data management. This practice is rooted in the ethical principle of non-maleficence, which focuses on minimising harm. Informed consent is the foundational principle for sharing individual data. Allen et al. underscore the existing gaps in evidence regarding the delegation of consent to LLMs. A key concern is the accuracy of the medical information provided by LLMs, which includes the risk of AI-generated misinformation, as demonstrated by the hallucinations. Strategies to mitigate such risks include implementing robust validation mechanisms, limiting AI-generated outputs to well-defined clinical frameworks, and delineating AI-generated content from verified clinical guidelines (34). These measures are crucial for safeguarding individuals' autonomy and ensuring that AI integration in digital health frameworks adheres to ethical principles.

### 2.6 The ethical challenge

Ethical issues related to accuracy, bias, patient confidentiality, and accountability are inherent in healthcare practices. Transforming medicine and enhancing wellbeing within digital health infrastructures demand a unified approach that addresses these ethical concerns through collaboration and interdisciplinary dialogue (35).

The only international legally binding instrument protecting human rights in the biomedical field is the Convention on Human Rights and Biomedicine (ETS No 164). The Convention aims to protect the dignity and identity of all human beings and guarantee everyone, without discrimination, respect for their integrity and rights and fundamental freedoms concerning the application of biology and medicine. A Strategic Action Plan (SAP) on Human Rights and Technologies in Biomedicine was adopted by the Committee on Bioethics during its 16th meeting in 2019 (36). This SAP addresses the emerging challenges posed by new genetics, artificial intelligence, and big data technologies, particularly when these elements are combined to create new applications. The emergence of these technologies raises ethical questions regarding autonomy, privacy, and non-discrimination. It emphasises the importance of ensuring equitable access to healthcare, stating that all individuals have the right to be informed about their health, including the results of predictive genetic tests (36). While AI is becoming increasingly adept at diagnostics, its reliance on vast amounts of personal data raises significant ethical concerns regarding data ownership, privacy, and the transparency of AI-generated recommendations. This issue is further complicated because AI algorithms may inadvertently perpetuate biases embedded in the training data, exacerbating disparities for marginalised groups.

It is noted that the role of governance in biomedicine often focuses on facilitating technology applications and mitigating emerging risks. Human rights considerations typically come into play only after technological applications are developed and established, often making these technological pathways irreversible (36). To address this issue, the Convention concludes that there is an urgent need to integrate human rights into technologies with biomedical applications. Innovative treatments and healthcare technologies must be accessible to everyone to prevent harmful inequities that disproportionately affect already disadvantaged individuals and groups (36). Implementing targeted measures to prevent discrimination and mitigate health disparities becomes essential as AI systems become more prevalent in clinical settings. State parties to the Convention must implement robust measures to prevent discrimination and ensure that new developments do not create or worsen existing disadvantages.

#### 2.6.1 Kant's philosophy

People with rare conditions are viewed as a distinct group in society. Their experiences are shaped by their medical conditions and the societal perceptions of those conditions. The moral principles of freedom of thought should be considered to establish ethical rules of conduct. Freedom of thought, defined as “knowing what we can know”, is rooted in Kant's *Critique of Pure Reason* (CPR) (37). Baquero's (37) review aims to introduce Kant's principles to the field of natural science, emphasising that knowledge is always partial. According to Kant, the correspondence between truth and reality is inherently incomplete (CPR A42/B59). When we observe something outside of ourselves, it is accompanied by inner thoughts. The inner mind is “receptive to the senses” (CPR A50/B74) and represents a conscious subject (a “me”) that can be influenced by external factors. These external elements consist of natural “things” that can affect our sensibility. However, these things remain unknowable in their intrinsic ontological nature. In Kant's terms, they are simply “things-in-themselves”, “intelligible existences”, or “noumena” (CPR, B306) (38). In other words, our perception of the world is shaped by how we observe and think about it. We cannot know the world as it truly is, because we can only experience it through our senses and thoughts. Our sensibility utilises time and space to filter and structure what we observe.

**Figure 1:**
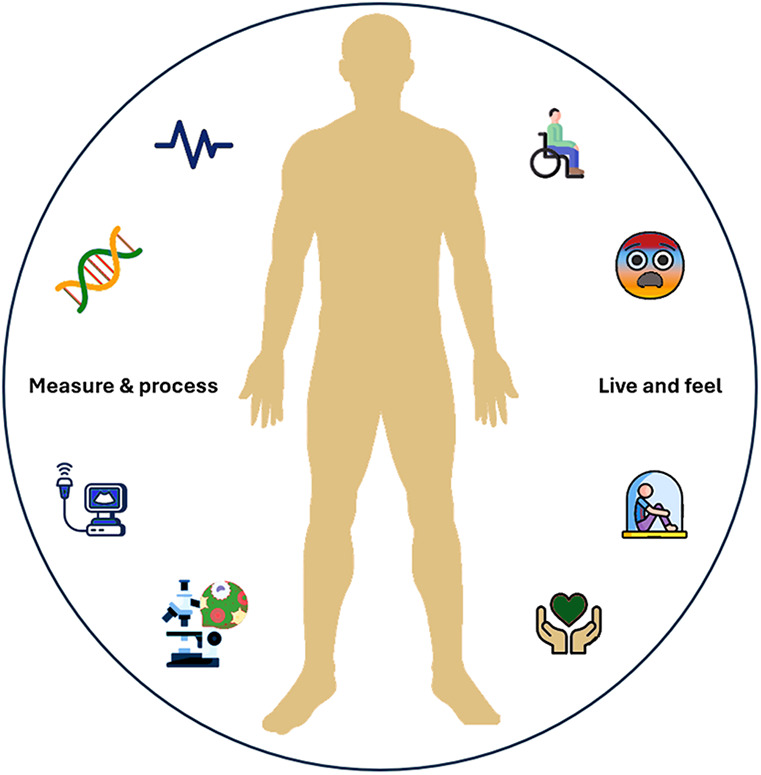
Seeing the whole person: “Live and feel” vs. “Measure and process”. A person with a chronic disabling condition is both “Live and feel” and “Measure and process.” Effective care requires understanding not only clinical data but also the lived, emotional, and social experiences of the individual.

In the context of rare diseases, this concept underscores the inherent subjectivity in interpreting health-related experiences. Each individual has their own perception of reality. This suggests that, since we perceive the world through our senses, reality varies from person to person and changes with time and place. Some aspects of our experiences remain unknowable; in Kant's terms, they are “things in themselves” (Dinge and Sich). People with genetic conditions often struggle with the lack of public understanding regarding their lives. Each condition affects individual senses and thoughts differently, and those without the same condition cannot fully grasp these unique experiences. For instance, individuals with Huntington's disease often experience movement disorders, cognitive decline, and emotional instability as a consequence of the genetic mutation. Nevertheless, their lived experiences of these symptoms extend beyond observable clinical manifestations, encompassing personal narratives that only they can convey (Figure 1). Similarly, while healthcare providers understand the genetic basis of Down syndrome and its associated medical risks, they cannot fully comprehend what it means to live with an extra chromosome 21 as an experience shaped by complex social, emotional, and cognitive dimensions.

This disconnect highlights a critical ethical consideration. The general public needs to understand that only individuals with chronic disabling conditions can genuinely convey the reality of living with those conditions. For others, people with rare diseases can become subjects of curiosity for researchers, with their data leveraged for scientific advancement, or their data collections are profitable for industry as a commercial gain, often without meaningful representation in decision-making processes.

Therefore, adhering to the principles outlined in the Convention is essential. It calls us to “protect the dignity and identity of all human beings”. Individuals with chronic disabling conditions should play an active role in all decisions affecting their lives, including preventive measures, management, and social support following a diagnosis, as well as developing high-quality digital solutions. Kant's philosophy serves as a guiding principle for integrating personal experiences into digital health frameworks, advocating for a person-centred approach that respects individual autonomy and acknowledges the inherent limitations in capturing the full reality of rare disease experiences.

The illustration depicts the fundamental challenge in rare disease care, drawing on Kant's distinction between the measurable clinical data that medical systems can capture (elektro-encefalogram, biomarkers, scans, tests) and the unmeasurable lived experience of patients (pain, fatigue, fear, hope, dignity). Effective care requires bridging both realms - acknowledging that while we can never fully quantify a patient's inner reality, optimal care must somehow account for both the observable phenomena we can study and the unknowable experience patients actually live.

## 3 Discussion

Properly implemented digital transformation of healthcare data enhances medical and social care using real-world data collected from EHRs, including data from medical devices, personally reported ICF questionnaires and other sources. In this real-world data ecosystem, the scale of data resources must be harmonised to become standard routine markers and endpoints.

A critical component of this harmonisation is the implementation of whole-genome variant profiling as a standard part of healthcare. Systematically integrating genomic data will help prevent the comorbidity associated with chronic and rare conditions, aligning preventive care strategies with genetic risk profiles. However, integrating genomic data also raises ethical considerations regarding individual privacy, data ownership, and informed consent, particularly when AI systems are involved in data analysis and interpretation.

As the digital health landscape evolves, patients increasingly seek comprehensive interactive access to their health records through user-friendly and high-quality apps or portals. This interactive access empowers patients and their families to make informed decisions and facilitates the timely sharing of critical health information across clinical and social care settings.

However, internet resources currently do not adequately facilitate personal data input. Clinicians, managers, and policymakers need to recognise that the advantages of providing such access far outweigh the associated risks (39). Addressing person-centred care is a moral term as we explore the potential of digitalisation to improve healthcare delivery. The importance of defining clinical data points is illustrated by comparing data gathered by manually selecting data points from the clinical guidelines with GPT4's advanced artificial intelligence systems. Thus, human insights regarding the quality of data and clinical relevance must be prioritised before using AI with these data. The dual approach- leveraging AI for efficiency while maintaining human oversight for accuracy- represents a balanced strategy for digital health integration.

## 4 Conclusion and recommendations

(For policymakers, health professionals, and developers; and priority areas for future research or policy development):

### 4.1 Rare diseases as an identity

People with a rare conditions encounter diagnostic failures, uncertainty about treatment options, and various disabilities that affect their health, psychosocial wellbeing, and economic circumstances. A holistic person-centred approach addressing the needs of persons living with a rare disease fulfulling human rights to overcome the barriers they face, is aphoristic. It is not acceptable that 58% experience discrimination related to the rare disease or disability in healthcare, housing, employment and education. Policymakers and healthcare providers should be made aware of these evidencebased patient reported facts.

### 4.1 Public policies

The UN and the WHO have addressed the challenges of persons living with a rare disease and their families. We urge member states to act accordingly and strengthen “health systems, particularly in primary healthcare, to ensure universal access to a wide range of affordable and high-quality healthcare services for persons living with a rare disease, especially children”. Establishing more inclusive governance in digital health to help build trust in public health policies, including the digital transformation of health by adopting AI. The guiding principle of “data first, AI later” is fundamental to this vision. Research and training programs for healthproviders is recommended to understand how they can contribute to interopeable quality data based on their care practice and guidelines.

### 4.3 Era of digital health

People with a rare diseases often lack comprehensive digital data frameworks, leading to gaps in patient care facilitating full interactive access to real-life data. Personal data sharing would mean a significant cultural shift in healthcare. This shift allows health providers to dedicate more time to those who require the most medical and social support.

Digital expansion must not miss the unique opportunity to utilize the ICF framework. To enhance the well-being of people with rare conditions and their families, they should be enabled to define special needs with ICF core sets adjusted to specific conditions.

Large language models rely on existing text found on the internet, which often reflects the interests of researchers and profit-driven institutions. Collaboration among life sciences, information technology specialists, and patient representatives is essential for building public trust in digital health and artificial intelligence.

### 4.4 Ethical challenge

Patient-reported outcomes and lived experiences are crucial for developing accurate, person-centred digital health frameworks. Digital data on social participation are vital to ensure person-centred health services, particularly for poorly understood conditions.

Individuals with rare chronic disabling conditions provide valuable insights based on their perception of reality as the effect of biology and experience varies in time and place. They should be actively involved in all decisions about their care and data exchange, either as groups or individuals.

Research on digital literacy and training programs for people with a rare condition is a precondition.

To drive innovation, it is essential to incorporate the human perspectives on how digitalisation can enhance health and wellbeing.

## 5 Limitations

We understand this is a review of a complex theme and has limited selected literature. The reliance on a single case study may limit the generalizability of findings to other rare diseases. Still, from experience, we understand that the case illustrated issues are observed by many European patient organisations. However, there is potential bias due to the reliance on expert interpretation.
